# Wheeze and cough measurements at night in children with respiratory symptoms

**DOI:** 10.1186/s12887-020-02455-5

**Published:** 2020-12-12

**Authors:** Markus Lindenhofer, Lena Roth, Clemens Mädel, Florian Götzinger, Katharina Kainz, Christiane Lex, Thomas Frischer, Matthias Reinweber, Angela Zacharasiewicz

**Affiliations:** 1 Klinikum Favoriten, Wiener Gesundheitsverbund, Wien, Austria; 2 Wilhelminenspital, Klinikum Ottakring, Department of Pediatrics and Adolescent Medicine, Teaching Hospital of the University of Vienna, Montleartstrasse 37, 1160 Wien, Austria; 3 Department for Pediatric Cardiology and Intensive Care Medicine, University Hospital Göttingen, Göttingen, Germany; 4 Faculty of Medicine, Sigmund Freud University, Vienna, Austria; 5 Vienna Hospital Association, Healthcare Management, Vienna, Austria

**Keywords:** Cough, Wheeze, Cough monitor, Nocturnal symptoms, Children

## Abstract

**Background:**

Nocturnal cough and wheeze are important symptoms when diagnosing any respiratory disease in a child, but objective measurements of these symptoms are not performed.

**Methods:**

The aim of our study was to analyze the use of an automated detection system to assess breath sounds objectively in comparison to cough and wheeze questionnaires and to evaluate its feasibility in clinical practice.

**Results:**

Forty-nine recordings of thirty-nine children were processed (asthma *n* = 13; cystic fibrosis *n* = 2; pneumonia *n* = 5; suspicion of habit cough *n* = 7; prolonged, recurrent or chronic cough *n* = 13), and cough and asthma scores were compared to the objective nocturnal recordings. Time for audio-validation of recordings took between 2 and 40 min (mean: 14.22 min, (SD): 10.72). Accuracy of the automated measurement was higher for cough than for wheezing sounds. Nocturnal cough readings but not wheeze readings correlated with some of the corresponding scores.

**Conclusion:**

To our knowledge this is the first study using a new device to assess nocturnal cough and obstructive breath sounds objectively in children with a wide variety of respiratory diseases. The assessment proved user friendly. We obtained additional information on nighttime symptoms, which would otherwise have remained obscure. Further studies to assess possible diagnostic and therapeutic benefits of this device are needed.

## Background

Respiratory symptoms, such as coughing, are among the most common reasons being presented in pediatric primary care [1, 2]. Getting reliable information from patient or parents on nocturnal symptoms like cough or wheeze is challenging [3]**.** Often, the parents’ perception of the frequency of their children’s cough is not accurate [4]. Smoking parents tend to underestimate their offspring’s respiratory symptoms [5]. While for nocturnal cough both under- and overreporting has been noticed [4], truly little is known about the true incidence of wheezing or coughing during the night. Furthermore, different treatment approaches in children with wheezing may show benefit, if initiated early [6]. There are numerous reasons for cough and wheezing at night in children, and irrespective of the underlying diagnosis, symptoms are usually not objectively recorded. Hence, important information on the patient’s history is missing and only assessed by self-reporting or caregiver ‘s subjective perception.

In previous studies it has been shown that up to a third of children with asthma are suffering from nocturnal wheeze, with great improvement once a sufficient therapy has been established [7, 8]. Even asthmatic patients, who describe themselves as symptom-free may show wheeze if monitored objectively [9]. Consequently, negative effects of those undetected and hence untreated symptoms on the patients’ quality of life may occur [10–12]. Especially during COVID 19 pandemic uncontrolled asthma is an important risk factor for severe COVID-19 disease [13]. Thus, nocturnal wheeze, if detected early, should initiate appropriate therapy [14]. The Task Force of the European Respiratory Society on asthma monitoring concludes that different types and stages of asthma require different types of monitoring schemes [15]. While various biomarkers are currently used on asthma control, further investigations are needed on the impact of nocturnal cough and wheeze [15–18].

Acute pneumonia is an infection of the pulmonary interstitium and the alveoli. Fever, cough and/or shortness of breath are common symptoms with frequency and severity of cough differing between individuals and changing over the time course of the disease [19]. Little information is currently available on nocturnal cough frequency in children with pneumonia.

Cystic fibrosis (CF) is an inherited autosomal recessive disorder, mostly diagnosed at young age due to expanding newborn screenings [20] with clinical manifestation differing in individuals. However, lung disease usually is present from the early life [21] and a vicious circle of infection and inflammation in the lung causes pulmonary exacerbations resulting in progressive deterioration of lung function [22].

In children prolonged, recurrent or chronic cough can be the symptom of many underlying respiratory of systemic diseases. Knowledge of the objective quantity of the symptoms cough and wheezing at night may provide useful information both for the decision making for appropriate diagnostic measurements and necessary therapeutic agents [23].

Psychogenic cough mostly presenting with dry cough is usually non-existent at night [24, 25] and the diagnosis is made after excluding all other causes of chronic cough [23, 26].

Questionnaires for patients and parents [14, 27] have been developed to evaluate nocturnal, respiratory symptoms, and have shown partially good results. Still, compared to objective methods they are inconsistent [28]. Devices like the Leicester Cough Monitor or the VitaloJak offer an objective display of cough frequency [29–31], but none of these devices are used to detect nocturnal wheezing [32]. Our intention was to assess cough and wheezing at night in children suffering from various both acute and chronic respiratory diseases using a new automated detection device.

The LEO-Sound System may offer the possibility to perform objective and repeatable measurement of cough and wheezing frequency [33–38].

Therefore, the rational and aim of this study was to assess this new method of collecting additional information about the patients’ nighttime symptoms of cough and wheeze by firstly comparing audio recordings with questionnaire data and secondly by assessing the overall feasibility of this method in clinical practice.

## Methods

### Subjects

Both in- and out-patients aged 4 to 18 were enrolled for this study. Inclusion criteria were current respiratory symptoms such as cough, wheeze or shortness of breath. We excluded patients, who were respiratory unstable and needed intensive care and those with the detection of multiresistant pathogens, such as methicillin-resistant *staphylococcus aureus* (MRSA), oxacillin-resistant *staphylococcus aureus* (ORSA), vancomycin-resistant *staphylococcus aureus* (VRSA), glycopeptide-resistant enterococci (GRE), vancomycin-resistant enterococci (VRE), multidrug resistant Gram-negative bacteria (MRGN) and extended-spectrum beta-lactamase (ESBL). The study was approved by the Local Ethics Committee and written informed consent was obtained from participating children, adolescents and caretakers.

### Study design

All recordings using the Leo-Sound monitor were done overnight in an inpatient or outpatient setting, during 8 h of night sleep in the first 29 patients and for 10 h in the remaining 10 patients, due to getting the experience of higher practicability with longer recording time. The monitor was attached to each patient by medically trained personal. In the outpatient setting, recordings were performed at home and the devices were returned to the hospital for analyses. Current symptoms were evaluated before and after the measurement by medical doctors, using validated scores and questionnaires (see below). The Leo-Sound-Analyzer (Löwenstein Medical GmbH & Co. KG, Bad Ems, Germany) analyzes recordings automatically. All breath sounds, which were classified as cough or wheeze by the Leo-Sound Analyzer_,_ were thereafter manually checked for accuracy and plausibility by a medically trained observer listening to the detected sounds. For continuity, the same two observers validated the recordings. Time needed for audio-validation of the recordings was noted.

### Leo-Sound

**Fig. 1:**
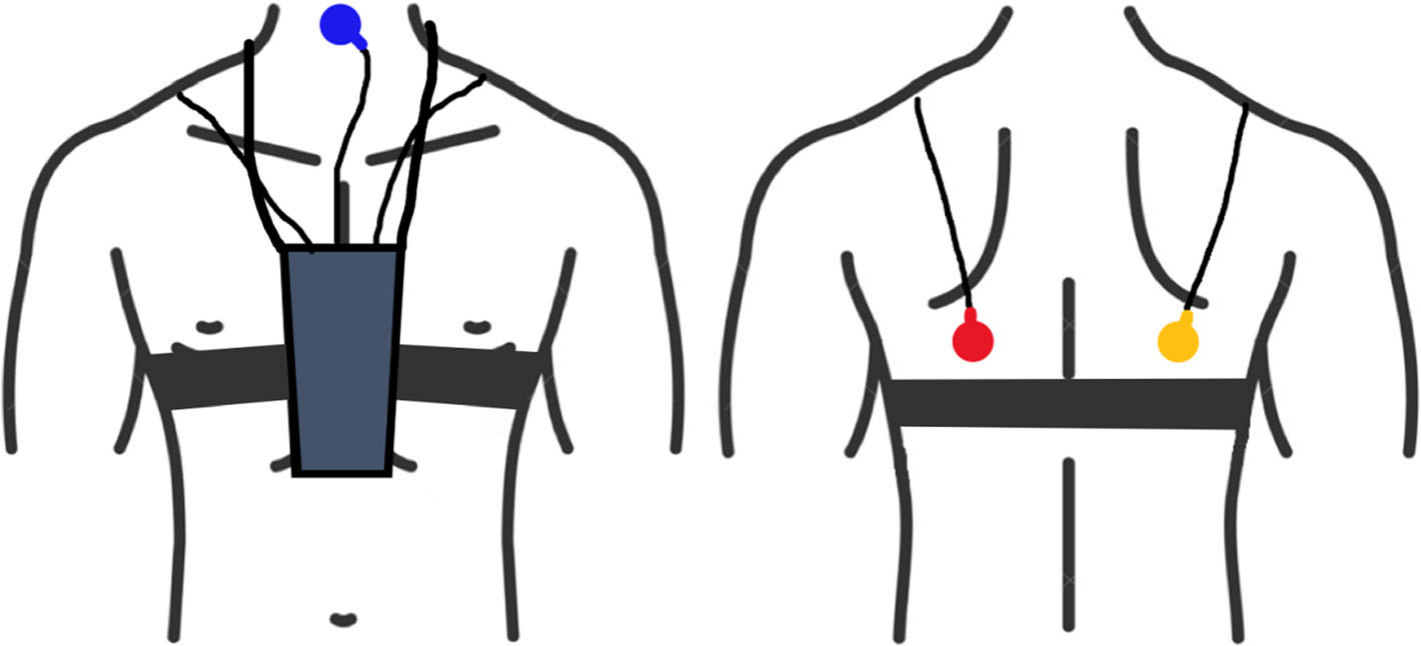
Setup of the Leo-Sound monitor and its bioacustic microphones. The blue microphone positioned on the trachea, the red and yellow one positioned on the right and left lung. Auscultation was used to find the ideal position for the sensors on the back

The Leo-Sound Lung-Sound-Monitor_®_ (Löwenstein Medical GmbH & Co. KG, Bad Ems, Germany) is a 35 mm × 75 mm × 170 mm (H x W x L) big device that was developed to objectively record and analyze cough and wheezing for a maximum duration of 24 h. Three microphones were placed on the skin in the area of the trachea – for distinguishing cough sounds – and both lungs to record the patient’s respiratory sounds (Fig. 1). The spots for the two microphones on the back were chosen after auscultation was done on each patient. Results of this examination were not taken into account for analysis. After the measurement was completed the monitor was connected to an HP ProBook laptop and the recordings were automatically analyzed by the Leo-Sound-Analyzer_®_. The program searches for frequencies and amplitudes specific for cough and wheeze, respectively. Identified respiratory sounds were then illustrated on a timeline, afterwards, the sounds identified as cough and wheeze were then assessed for measurement validation manually. In the process of validating the automated analyses, sounds like snoring, harrumphs, talking by the patient, alarm signals of other monitors, background noise or bowel movement have been found to mimic coughing and wheezing, respectively. Data of both the absolute number of lung sounds and the amount of 30-s periods, in which lung sounds were identified, were obtained, before and after validation by an observer. False negative coughs and wheezes were not further assessed. It should be noted that the analyzing program did not distinguish between wheezing at inspiration and wheezing at expiration. Therefore, wheezing sounds were further assessed, and distinguished manually whether they occurred during inspiration or expiration. Both wheezing at inspiration and expiration as well as wheezing at expiration-only were analyzed.

### Questionnaires

**Fig. 2:**
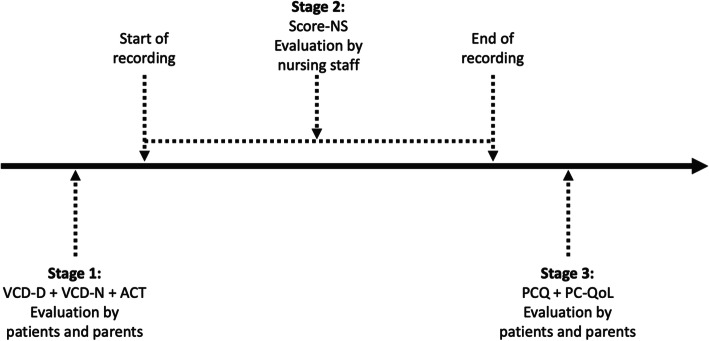
Time-line of the evaluation. The first evaluation was usually done just before the monitor and the microphones were applied on to the patient. The starting point of the recording was chosen individually for each child, accounting for their typical sleep schedule. Evaluations by the nursing staff naturally were only done in an inpatient setting. The last evaluation took place the morning after the recording, when the monitor was collected by staff. VCD-D = verbal category descriptive score for cough at daytime; VCD-N = verbal category descriptive score for cough at nighttime; ACT = Asthma Control Test; Score-NS = score for cough during recording as perceived by nursing staff (only in-patients); PCQ = pediatric cough questionnaire; PC-QoL = parent-proxy quality of life questionnaire for pediatric chronic cough

The evaluation by means of scores and questionnaires was done in three stages (Fig. 2). Firstly, before the recording, two VCD (verbal category descriptive) scores developed by Chang et al. [28] covering cough at daytime (VCD-D) and cough at nighttime (VCD-N) were used to evaluate severity and frequency of cough in our patients. Cough at daytime was evaluated to see if a correlation with nighttime symptoms could be observed. Only participants diagnosed with asthma were also given the Asthma Control Test (ACT) [39, 40] to determine asthma control over the last 4 weeks. Patient characteristics regarding age, gender, current medication and exposure to secondhand smoking were obtained. Additionally, FEV1 and the Tiffeneau index from prior lung function tests were obtained, if recent data was available**.**

The second stage took place during the recording: Children, who were assessed in an inpatient setting were evaluated additionally by the nursing staff in terms of the perception of cough and/or other breath sounds every 2 h, using a newly developed, simplified score (Score-NS) that has not yet been validated.

At stage three – after the recording – all caregivers/families who spent the night of the recording at home or on the ward with their children were given a simplified translated version of the validated pediatric cough questionnaire (PCQ) developed by Hartnick, et al. [41] to capture the parent’s perception of their children’s nocturnal symptoms during the recording. The last ten patients were also evaluated using the main parts of a validated parent-proxy quality of life questionnaire for pediatric chronic cough (PC-QoL), developed by Newcombe et al. [42], which was translated into German language.

### Statistical analysis

Depending on the variable’s distribution continuous data are either expressed as means and standard deviation (SD) or median and interquartile range (IQR) and were analyzed with multivariate analysis of variance (MANOVA) and Pearson-Correlation respectively. Categorical variables are expressed in absolute numbers and percentages. Normality-tests have been measured using the Kolmogorov-Smirnov test (KS-test) und homogeneity of variance was proofed using Levene’s test of homogeneity of variance. Posthoc-testing was performed using the Bonferroni corrected z test. Results were categorized as statistically significant with an alpha level set < 0.05 and < 0.01; the reported *p*-values are two-sided. All analyses were performed using SPSS, version 25.0 (IBM Corp, Armonk, NY, USA).

## Results

From 47 children matching the inclusion criteria 5 children declined to participate; three were excluded, because of low quality recordings, resulting in *n* = 39 children with valid measurements (23 males, 16 females; median age 9.53 years, range 4–17 years). Nine children had more than 1 recording and 1 statistical outlier resulting in 49 valid recordings. Nineteen recordings were obtained in an inpatient setting and 30 were done in an outpatient setting (Table 1). If two recordings were done on consecutive nights, the mean of both recordings was taken, as no significant difference between the two measurement nights had been found**.** All other recordings were analyzed individually. Finally, 43 recordings were used for the statistical analysis.

**Table 1:** Demographics of the study population for both recordings and patients

	Recordings (%)	Patients (%)
All	43 (100%)	39 (100%)
Gender		
Male	24 (55,8%)	23 (59%)
Female	19 (44,2%)	16 (41%)
Location of recording		
Inpatient	18 (41,9%)	N/A
Outpatient	25 (58,1%)	N/A
Age		
4–8	19 (44,2%)	16 (41%)
9–13	17 (39,5%)	16 (41%)
14–17	7 (16,3%)	7 (18%)
Secondhand smoking		
Yes	10 (23,2%)	10 (25,6%)
No	27 (62,8%)	26 (66,6%)

The data of 4 patients, who were recorded twice, but not on consecutive nights, were counted separately, making the difference between *n* = 39 patients and 43 valid recordings

Data from 20 lung function tests were available for analysis, including 9 sets of data were bronchodilation was used (Table 2). Nine patients had not been prescribed any medication at the time of the recording. All other participants had either been prescribed antibiotics, beta2-sympatiomimetica or inhaled glucocorticoids. The mean absolute number of coughs per child post validation was 18.78 (SD: 26.29) per recording. Furthermore, the mean amount of cough episodes (i.e. 30-s-periods in which at least one cough was registered) was 7.17 (SD: 9.31). The absolute number of wheezes per child post-validation was 488.72 (SD: 1048.40) per recording. The mean number of wheezing episodes (i.e. 30-s-periods in which at least one wheeze was registered) was 71.69 (SD: 130.38). Taking just expiratory-only wheezing into account the absolute number of wheezes per child post-validation was 71.34 (SD: 29.72). Some children did not register any cough or wheeze. 25,7% of all detected and validated obstructive respiratory sounds were identified as wheezing on expiration only - typical of obstruction within the lung and found in asthmatic children.

**Table 2:** Lung function data and medication of included patient groups

	Asthma	Habit	CF	Unknown	Pneumonia	All
Patients/recordings	13/14	7/7	2/3	13^a^/14	5^a^/5	39^a^/43
Lung function						
N=	11	4	3	7	1	26
FEV1 (L)	1,44	2,35	1,09	1,78	1,23	1,63
FVC (L)	2,17	2,36	1,56	2,04	1,51	2,07
FEV1/FVC (%)	68,45%	92,53%	67,67%	86,15%	81,00%	77,31%
After bronchodilation						
N=	8	1	0	1	1	11
FEV1 (L)	1,71	1,51	–	2,13	1,18	1,68
FVC (L)	2,33	1,66	–	2,29	1,47	2,19
FEV1/FVC (%)	72,70%	90,81%	–	93,05%	80,18%	76,88%
Medication						
Steroids	12	3	0	6	0	21
β-2 sympathomimetics	13	2	3	7	2	27
Antibiotics	1	0	0	1	4	6

This table shows the mean of lung function data for both, before and after bronchodilation, if available

*FEV1* forced expiratory volume per second, *FVC* forced vital capacity

^a^one patient was first included with acute pneumonia and later on again with chronic respiratory symptoms

Six different questionnaires to assess respiratory symptoms were used; these were subjective tools and 3 of the 6 questionnaire scores used in this study correlated with the cough recordings measured (Table 3). Firstly, the VCD-D, which examines cough at daytime (*r* = 0.46, *p* < .01), and secondly the VCD-N, which examines cough at nighttime (*r* = 0.42, *p* < .01), showed a significant correlation to objective cough recording data. There was also a significant correlation between cough recordings and the nursing staff score, Score-NS (*r* = .713, *p* < .05). The other validated scores – PCQ, the ACT and the PC-QoL – did not show any correlation with the measurements of nocturnal cough. Furthermore, we found no correlation of cough measurements with the lung function parameters FEV_1_ and the Tiffeneau index, current medication, recorded passive smoke exposure or other variables like age, gender, or the location of the recording. Finally, none of the questionnaires or other parameters correlated with the nocturnal wheezing recordings.

**Table 3:** Correlation detections with questionnaires and scores

		VCD-N	VCD-D	ACT	PCQ	PC-QoL	Score-NS
Coughing							
N=		35	34	17	19	7	12
Episodes with cough	r	0,48^**^	0,41^*^	0,05	0,07	−0,33	0,42
	*p*-value	0,00	0,02	0,85	0,76	0,47	0,17
Number of coughs	r	0,42^*^	0,46^**^	0,07	0,08	−0,26	0,71^**^
	*p*-value	0,01	0,01	0,79	0,73	0,56	0,01
Wheezing							
N=		34	33	17	19	7	11
Episodes with wheezing	r	0,21	0,11	−0,03	−0,07	−0,44	0,07
	*p*-value	0,23	0,52	0,89	0,78	0,32	0,84
Number of wheezes	r	0,22	0,08	−0,03	−0,07	− 0,43	0,08
	*p*-value	0,22	0,66	0,90	0,78	0,34	0,82
Wheezes at expiration only	r	0,11	0,15	0,06	-0,07	0,69	0,09
	*p*-value	0,56	0,43	0,81	0,78	0,13	0,78

*VCD-D* verbal category descriptive score for cough at daytime, *VCD-N* verbal category descriptive score for cough at nighttime, *ACT* Asthma Control Test, *Score-NS* score for cough during recording as perceived by nursing staff (only in-patients), *PCQ* pediatric cough questionnaire, *PC-QoL* parent-proxy quality of life questionnaire for pediatric chronic cough; *p*-value: two-tailed *p*-value of Pearson’s correlation; *: correlation between subjective score and objective recording results with *p* < 0,05; **: correlation with *p* < 0,01

**Fig. 3:**
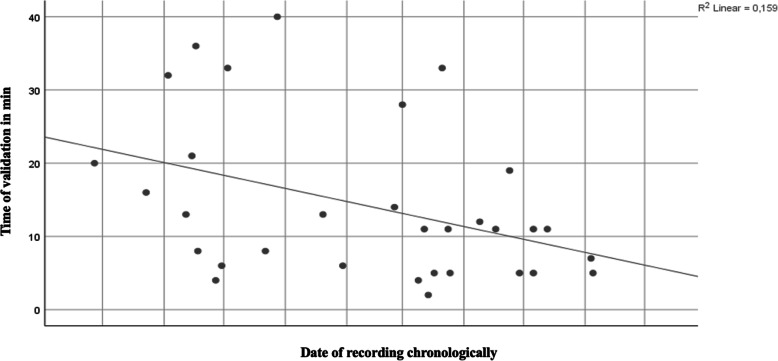
Time of manual validation. The time of manual validation of the recordings shown chronologically from the first (left) to the last (right)

Measurement of time to manually validate results was recorded in the first 29 patients. The average time for manual validation of a recording was 14.22 min (SD: 10.72 min), ranging from 2 to 40 min. A learning curve was detected (*r* = −.398; *p* < .05) (Fig. 3) and measurements were double checked independently by two doctors. The automatic-only-evaluation of breath sounds performed better at detecting cough (mean: 59.14%, SD: 33.13%; median: 71.11%, IQR: 36.3–86.3%) than at classifying wheezing sounds correctly (mean: 37.36%, SD: 35. 07%; median: 34.5%, IQR: 0–62.25%), meaning, only 59.14% of coughs and 37.36% of wheezing sounds were already interpreted correctly by the used analyzing program prior to manual validation.

## Discussion

In this study, firstly we demonstrate that the device used (Leo-Sound) can also measure obstructive respiratory sounds like wheeze during sleep setting it apart from other available cough recording devices. Cough monitors like the Leicester Cough Monitor or the VitaloJak have already shown promising results [32, 43], but are restricted to assessing cough, making wheezing again undetected. Secondly, we were able to show that results can be obtained in a user-friendly fashion with an acceptable time to obtain a validated result. The usefulness of the monitoring device was also demonstrated by Grosse-Onnebrink et al. in a study of patients with cystic fibrosis and primary ciliary dyskinesia [36]. Although manual validation was also done in this study, there was no information published on how well the automated analyzing program interpreted respiratory sounds. A study published by Koehler et al. compared subjective and objective assessments of cough in children with acute bronchitis, using the Leo-Sound, and showed a significant correlation between the two [38]. Contrary to those findings, only two of the previously validated scores used in our study correlated with the objective nighttime cough recordings. The VCDs (VCD-D, VCD-N) developed by Chang et al. [28], covering cough at day- and nighttime, respectively, are simple verbal descriptive scores using a scale from 0 to 5 to evaluate the severity of the cough and correlated with objective measurements. The other cough questionnaires did not correlate with the recorded and verified, nocturnal cough amount, demonstrating a poor agreement between subjective and objective assessment. However, overall numbers are small and therefore the results should be interpreted with caution, since the return number of the scores used, excluding the VCDs, was low.

In this study a new in-house score was used, utilizing very simplified evaluations by the nursing staff every 2 h, whether coughing was perceived or not. This score did correlate with the amount of nocturnal cough, however, the number of patients again was small (*n* = 11). Therefore, relying on the assessment of nocturnal cough by the nursing staff might be a good alternative to objective recordings, in an in-patient setting only. However, workload of hospital staff and background noise on any busy pediatric ward are limiting factors.

With regards to the measurements of wheezes, the well-established ACT score did not correlate with the results of nocturnal measurements of expiratory wheezing. Therefore, our results further underscore the importance of using objective tools to monitor nighttime symptoms. Nocturnal wheezing measured objectively did not correlate with lung function values of FEV_1_ or the Tiffeneau index, both before and after bronchodilation. However, again the results must be interpreted with caution due to the small number of patients. Furthermore, some of the lung function measurements were not performed within a few days to the nocturnal measurement. In 5 children time between the nocturnal measurement and lung function was 2–8 weeks, hence lung function might have been quite different on the day of the nocturnal measurement. In our study, about 40% of coughing events and 60% of wheezing events were initially interpreted falsely by the automated program; this is in contrast to a study published in 2015 by Gross et al., describing the initial sensitivity and specificity for both cough and wheeze without re-listening to sounds manually after recording to verify them to be over 90% [37]. One explanation of the discrepancy was that background noise may have interfered with our measurements in some cases.

Possible obstacles for wider use in clinical practice may be the need to reassess the measurements manually to exclude false positive cough and wheeze events, which we have demonstrated in our study. With training manual validation can be achieved in less than 10 min, which we consider an acceptable time frame in the clinical setting. This is similar to manual checks done after long time measurement such as sleep studies, long term blood pressure of other physiological measurements.

One limitation of our study is the fact, that we were not able to include a control group into our study for comparison. Another limitation of the study is the small sample size and the heterogeneity of the study population. Nevertheless, we have shown that the method described can be used in various pediatric respiratory diseases. Although different separate diagnostic groups are too small to make significant claims about benefits for the patients, our findings are promising. In habit cough patients for instance, demonstration of complete lack of cough during nighttime sleep, confirmed suspected diagnosis in these cases. If and to what extend a recording’s result would affect a child’s treatment or diagnosis, should thus be subject of future studies.

Possible future areas of use may be the adjustment of anti-inflammatory therapy in asthmatic patients and children suffering from various wheezing phenotypes in particular if they are symptomatic at night [44]. Also, treatment optimization in patients with cystic fibrosis and the diagnosis of patients suspected to suffer from psychogenic cough might be possible. Further studies on indication and benefit from the procedure are necessary.

## Conclusion

In summary, we have shown a discrepancy between nocturnal measurements and most subjective symptom reports by questionnaires. Nocturnal breath sound monitors like the Leo-Sound device used here, seem to be useful measurement tools to objectively assess nocturnal respiratory symptoms in children.

## Data Availability

The datasets used and/or analyzed during the current study are available from the corresponding author on reasonable request.

## Abbreviations

CF: Cystic fibrosis
FEV1: Forced expiratory volume per second
FVC: Forced vital capacity
VCD-D: Verbal category descriptive score for cough at daytime
VCD-N: Verbal category descriptive score for cough at nighttime
ACT: Asthma Control Test
Score-NS: Score for cough during recording as perceived by nursing staff (only in-patients)
PCQ: Pediatric cough questionnaire
PC-QoL: Parent-proxy quality of life questionnaire for pediatric chronic cough
SD: Standard deviation
IQR: Inter quartal range
MRSA: Methicillin-resistant *staphylococcus aureus*
ORSA: Oxacillin-resistant *staphylococcus aureus*
VRE: Vancomycin-resistant *staphylococcus aureus*
GRE: Glycopeptide-resistant enterococci
VRE: Vancomycin-resistant enterococci
MRGN: Multidrug resistant Gram-negative bacteria
ESBL: Extended-spectrum beta-lactamase
